# Effect of Phenolic Compounds from Elderflowers on Glucose- and Fatty Acid Uptake in Human Myotubes and HepG2-Cells

**DOI:** 10.3390/molecules22010090

**Published:** 2017-01-06

**Authors:** Giang Thanh Thi Ho, Eili Tranheim Kase, Helle Wangensteen, Hilde Barsett

**Affiliations:** 1 Department of Pharmaceutical Chemistry, School of Pharmacy, University of Oslo, P.O. Box 1068 Blindern, 0316 Oslo, Norway; helle.wangensteen@farmasi.uio.no (H.W.); hilde.barsett@farmasi.uio.no (H.B.); 2 Department of Pharmaceutical Biosciences, School of Pharmacy, University of Oslo, P.O. Box 1068 Blindern, 0316 Oslo, Norway; e.t.kase@farmasi.uio.no

**Keywords:** elderflower, *Sambucus nigra*, type 2 diabetes, human skeletal muscle, HepG2-cell, metabolic disorder, antioxidant, glucose uptake, oleic acid uptake

## Abstract

Type 2 diabetes (T2D) is manifested by progressive metabolic impairments in tissues such as skeletal muscle and liver, and these tissues become less responsive to insulin, leading to hyperglycemia. In the present study, stimulation of glucose and oleic acid uptake by elderflower extracts, constituents and metabolites were tested in vitro using the HepG2 hepatocellular liver carcinoma cell line and human skeletal muscle cells. Among the crude extracts, the 96% EtOH extract showed the highest increase in glucose and oleic acid uptake in human skeletal muscle cells and HepG2-cells. The flavonoids and phenolic acids contained therein were potent stimulators of glucose and fatty acid uptake in a dose-dependent manner. Most of the phenolic constituents and several of the metabolites showed high antioxidant activity and showed considerably higher α-amylase and α-glucosidase inhibition than acarbose. Elderflower might therefore be valuable as a functional food against diabetes.

## 1. Introduction

Type 2 diabetes (T2D) is one of the most prevalent and serious metabolic diseases, characterized by hyperglycemia, hypertension, dyslipidemia and obesity [1]. Chronic hyperglycemia is caused by insulin resistance, pancreatic β-cell failure and enhanced gluconeogenesis in the liver. Discovery of new hypoglycemic drugs with high potency and few or no side effects is important to improve quality of life for patients with T2D [2]. Recently, there has been increased interest in plants rich in phenolic compounds with antidiabetic properties. Elderflowers (*Sambuci flos*), the flowers of *Sambucus nigra* L. (*Adoxaceae*), are widely used in traditional medicine for treatment of respiratory diseases, influenza, inflammation, as a diuretic and for relieving symptoms and conditions related to what we know in modern days as diabetes [3,4]. Chemical and pharmacological investigations have revealed that elderflowers contain a variety of natural products such as flavonoids, tannins, pectins, triterpenoids and phenolic acids, which possess diverse biological activities such as anti-inflammatory, antioxidant, and anti-hyperglycemic effects [5,6,7,8].

The skeletal muscle and the liver play an important role in blood glucose control, storage and utilization of glucose [9]. T2D is associated with excessive free fatty acid (FFA) in plasma, prolonged physical inactivity, insulin resistance and/or systemic hyperlipidemia. High concentrations of plasma FFA are associated with increased risk for cardiovascular diseases. Thus, substances that stimulate glucose and unsaturated FFA uptake in the skeletal muscles and liver might play an important role in the pathogenesis of insulin resistance. Extracts from elderflower have shown increased glucose uptake, glucose oxidation and glucogenesis in rat abdominal muscle and to have a stimulatory effect on insulin secretion [8]. In previous studies dichloromethane (DCM) and methanol (MeOH) extracts from elderflowers and selected metabolites increased the glucose uptake in primary porcine myotubes, reduced fat accumulation in the nematode *Caenorhabditis elegans* and activated the human peroxisome proliferator activated receptor (PPAR)γ [10,11,12]. One important therapeutic approach for treating diabetes is to control postprandial hyperglycemia. The control of postprandial hyperglycemia is critical in the early therapy for diabetes. To delay the digestion of carbohydrates and the absorption of glucose through inhibition of the carbohydrate-hydrolyzing enzymes α-amylase and α-glucosidase, in the digestive tract, is one way to decrease the postprandial hyperglycemia [13]. An imbalance between antioxidants and free radicals may result in oxidative stress. The occurrence of oxidative stress in diabetes has been extensively documented [14,15,16]. 15-Lipoxygenase (15-LO) and xanthine oxidase (XO) are peroxidative and prooxidative enzymes, respectively, and sources of reactive oxygen species (ROS) in vascular cells and are involved in free radical production in diabetes [16]. Given the fact that oxidative stress plays a role in the development of complications in diabetes, substances that both prevent free radical formation and inhibit the production of ROS, as well as have an impact on glucose absorption and result in enhanced glucose uptake in muscle cells, may have clinical significance in diabetes therapy.

The main objective of this study was to investigate the influence on glucose and oleic acid uptake in human skeletal muscle cells and human liver cells by elderflower compounds. Due to the fact that polyphenols are metabolized to simpler phenolic substances [17], it was also of interest to investigate whether these metabolites could have an effect. In order to get more information about compounds in elderflowers as potential antidiabetic agents and their possible impact on oxidative stress, inhibition of the enzymes α-amylase, α-glucosidase, 15-LO and XO, and the scavenging of 1,1-diphenyl-2-picrylhydrazyl (DPPH) radical were tested.

## 2. Results and Discussion

### 2.1. Uptake of Glucose in Human Skeletal Muscle Cells and HepG2-Cells

**Figure 1:**
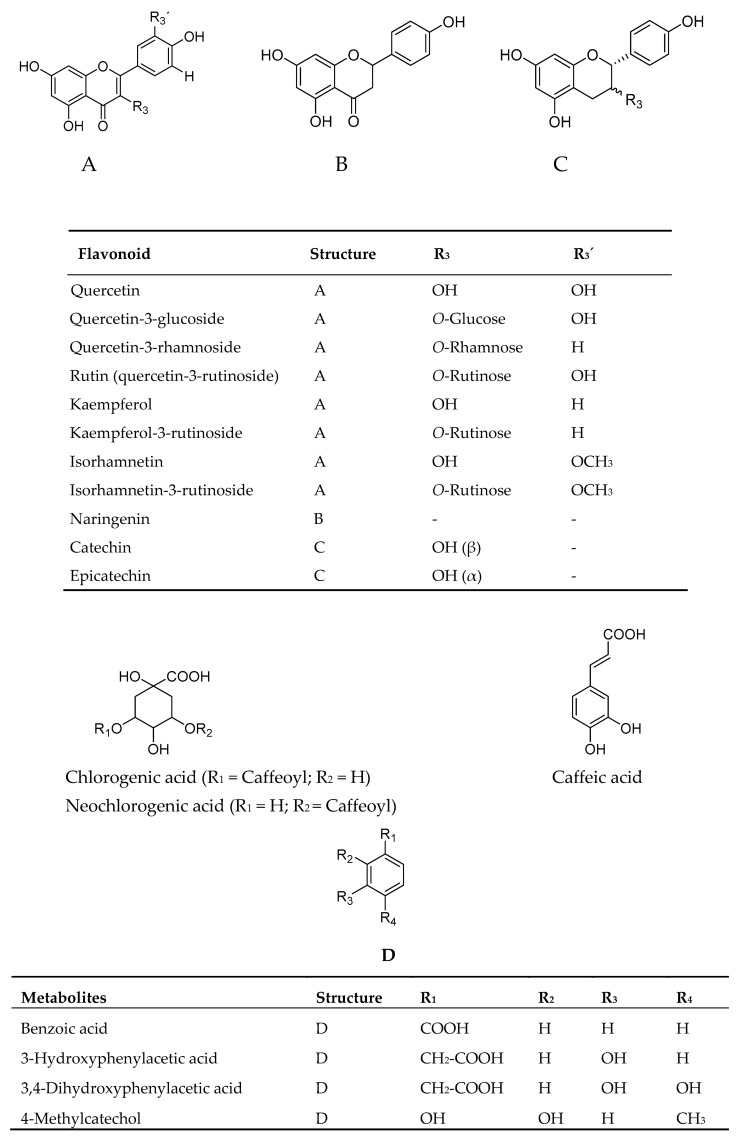
Structures of constituents and metabolites from elderflower.

**Figure 2:**
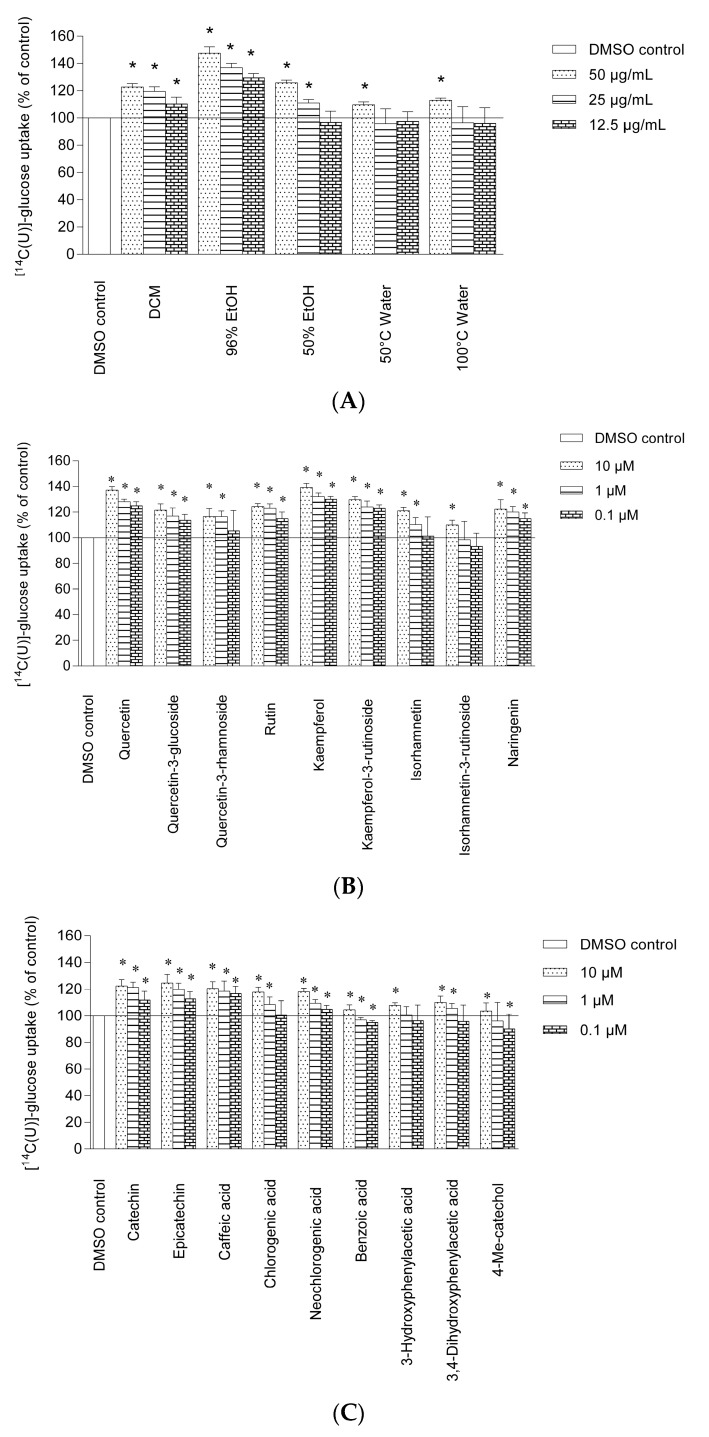
Effects of elderflower extracts, constituents and metabolites on glucose uptake in human myotubes. Myotubes were treated with (**A**) 12.5, 25 and 50 µg/mL of different crude extracts; (**B**,**C**) 0.1, 1 and 10 µM of elderflower phenolic compounds and metabolites for 2 days. Thereafter, the cells were exposed to [^14^C(U)]-glucose (1 μCi/mL, 100 μM) for 4 h as described in the Materials and Methods section. 22-*S*-hydroxycholesterol (22-SHC) (10 μM) was used as positive control. The figures show [^14^C(U)]-glucose uptake given as means ± standard error of the mean (SEM) (*n* = 3) from separate experiments. * *p* < 0.05 vs. control (0.1% dimethyl sulfoxide (DMSO)).

In the present study elderflower extracts, constituents and metabolites were investigated for their stimulation of glucose uptake in human skeletal muscle cells and HepG2-cells. Structures of elderflower constituents and metabolites that are included in this study are shown in Figure 1. Elderflower extracts, constituents and metabolites were investigated for their stimulation of glucose uptake in human skeletal muscle cells (Figure 2A–C) and HepG2-cells.

Several of the elderflower crude extracts (12.5 µg/mL–50 µg/mL) showed an increased uptake of glucose in a dose-dependent manner in human skeletal muscle cells and human liver cells as compared to 0.1% dimethyl sulfoxide (DMSO) control. The phenolic-sulfuric acid method [18] and ^1^H-NMR analysis revealed that high molecular weight carbohydrates were present in the 50% EtOH, 50 °C and 100 °C water extracts, while minor signals in the 50% EtOH extract ^1^H-NMR spectrum indicated the presence of caffeoyl moieties [19]. The ^1^H analyses also showed signals from aromatics protons, organic acids and monosaccharides in the 96% EtOH crude extract. Major signals could be attributed to rutin [20] and caffeoyl moieties [19], structures previously reported to be major constituents in the elderflowers [21]. Among the crude extracts the 96% EtOH (47.5% ± 4.7%) showed the highest increase, followed by the 50% EtOH (25.8% ± 3.5%) and the DCM extract (22.8% ± 2.4%) at 50 µg/mL (Figure 2A). The 50 °C and 100 °C water extracts only showed a significant increase at the highest concentration (50 µg/mL) (Figure 2A). Similar effects were also observed in the HepG2-cells for the crude extracts. The only exception was the 96% EtOH extract, at the highest tested concentration (50 µg/mL), which showed an increase in glucose uptake by 56.2% ± 3.1% in HepG2-cells compared to 47.0% ± 4.5% increase in the skeletal muscle cells. Elderflower constituents and metabolites exhibited an increase in glucose uptake in the skeletal muscle cells which are illustrated in Figure 2B,C. Among the flavonoids, kaempferol and quercetin showed the highest increase at 10 µM (39.1% ± 5.8% and 37.1% ± 4.6%, respectively). The maximal increase observed in the HepG2-cells was 45.2% ± 2.6% and 41.1% ± 3.2%, respectively. Kaempferol and quercetin were previously found to increase the glucose uptake in 3T3-L1 adipocytes [22]. In a separate study kaempferol was reported to increase the glucose uptake in HepG2-cells and in porcine myotubes in a concentration dependent way [10,23]. However, quercetin was observed to be ineffective in the same study [23]. The different cell types and experimental conditions used might be the explanation for the inconsistency in the effects of quercetin found in these two studies. The flavanone naringenin (0.1–10 µM) has been found to increase the glucose uptake in porcine myotubes and also to activate PPARγ [10,11]. This reported dose-dependent glucose uptake corresponds with our findings although different cell lines were used. Isorhamnetin-3-rutinoside, the glycosylated form of isorhamnetin, had a maximal increase of 10.0% ± 3.6% at 10 µM, while the aglycone isorhamnetin had an increase of 18.8% ± 4.3%. Glycosylated form seemed to have a lower increase of glucose uptake compared to the corresponding aglycone. The same trend was also observed for kaempferol and the glycosylated kaempferol-3-rutinoside with an increase of 39.1% ± 5.8% and 29.7% ± 2.5%, respectively, at the highest concentration tested, and for quercetin compared to rutin. Similar effects were also observed in the HepG2-cells. Among the flavonoid glycosides, rutin showed the highest increase of glucose uptake. Rutin is present in high amounts in elderflower [21] and may therefore be the most important contributor to the enhanced uptake of glucose by elderflowers. However, it is known that the bioavailability of rutin is quite low [24], it is hydrolyzed into its aglycon, quercetin, by colonic microbiota which may be absorbed and further metabolized [25]. Colonic bacteria also degrade flavonoid aglycones into smaller phenolic entities. 3-hydroxyphenylacetic acid, 3,4-dihydroxyphenylacetic acid and 4-methylcatechol, which are the degradation products of the flavonoid glycosides common in elderflowers [26] showed a small increase at the highest concentration tested (10 µM). Benzoic acid was inactive at 0.1 and 1 µM compared to DMSO control. This study revealed that the elderflowers, constituents and their metabolites are capable of enhancing glucose uptake in both skeletal muscle cells and liver cells with the flavonoid aglycones as the most promising compounds.

### 2.2. Uptake of Oleic Acid in Human Skeletal Muscle Cells and HepG2-cells

**Figure 3:**
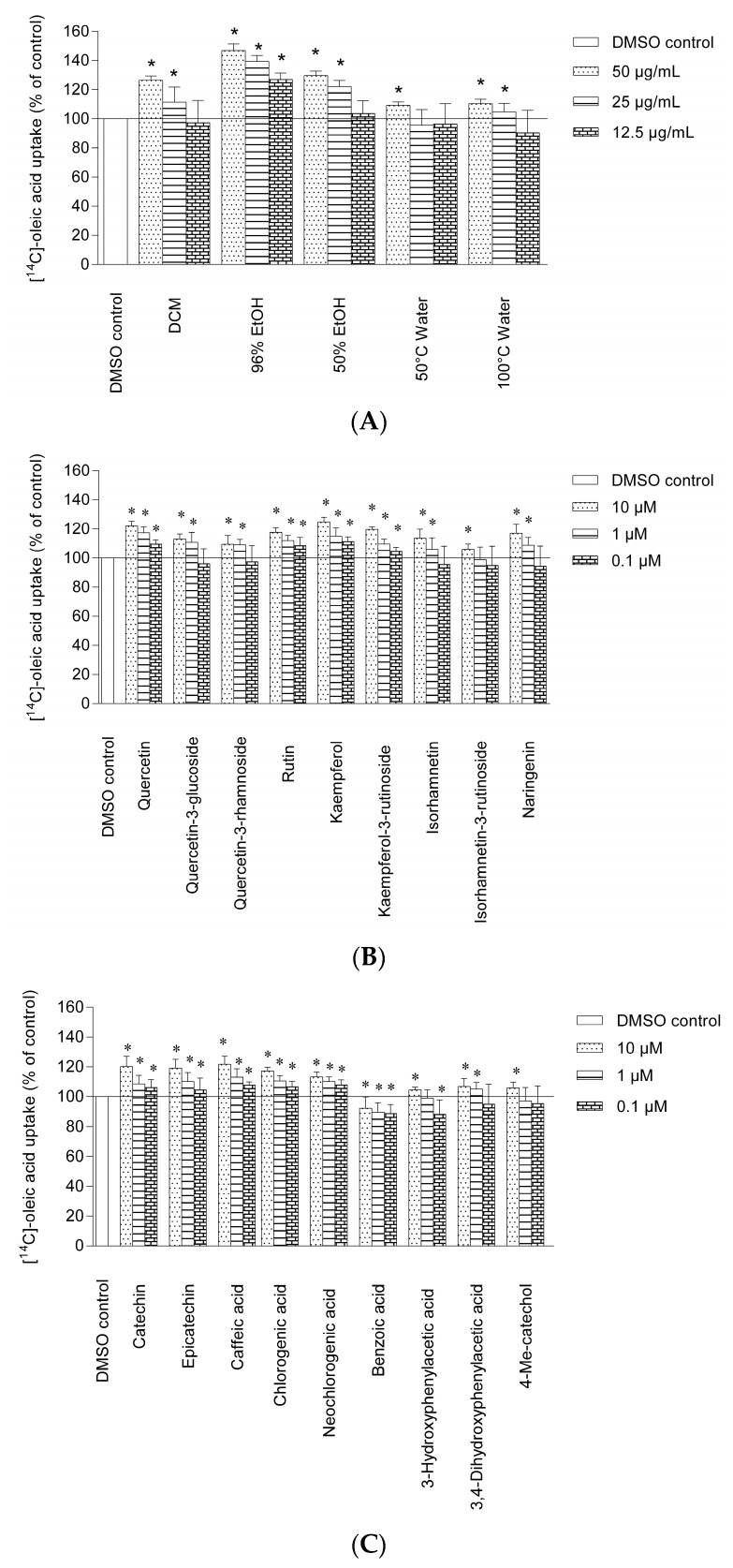
Effects of elderflower extracts, constituents and metabolites on oleic acid uptake in human myotubes. Myotubes were treated with (**A**) 12.5, 25 and 50 µg/mL of different crude extracts; (**B**,**C**) 0.1, 1 and 10 µM of elderflower phenolic compounds and metabolites for 2 days. Thereafter, the cells were exposed to [^14^C]-oleic acid (37 kBq, 100 µM) for 4 h as described in Materials and Methods. A synthetic liver X receptor (LXR) agonist T0901317 (10 μM) was used as positive control. The figures show [^14^C]-oleic acid uptake given as means ± SEM (*n* = 3) from separate experiments. * *p* < 0.05 vs. control (0.1% DMSO).

Polyunsaturated fatty acids and monounsaturated fatty acids have received a lot of attention due to their health benefits [27]. High consumption of oleic acid which is a monounsaturated omega-9 fatty acid reduced the risk of heart disease, cardiovascular disease and cancer [27]. Oleic acid has also shown a beneficial effect on insulin sensitivity, adipocyte glucose transport and prevents T2D [27,28,29,30]. The uptake of oleic acid in human myotubes after exposure to crude extracts, constituents and metabolites are shown in Figure 3A–C.

The 96% EtOH extract showed the highest oleic acid uptake in the human skeletal muscle cells at 50 µg/mL (47.0% ± 4.6%) followed by the 50% EtOH (29.8% ± 5.3%) and the DCM (26.7% ± 2.9%) extracts (Figure 3A). The 50 °C and 100 °C water extract showed a small but significant increase at 50 µg/mL. The effect of the crude extracts on HepG2-cells showed a higher increase of oleic acid uptake compared to the skeletal muscle cells.

The 96% EtOH extract had a maximal increase of oleic acid uptake by 54.6% ± 3.4%, followed by 50% EtOH extract with 38.5% ± 3.4% and DCM extract with 31.4% ± 2.2% at 50 µg/mL in the HepG2-cells. The increase of oleic acid uptake in human skeletal muscle cells and HepG2-cells induced by rutin, quercetin-3-glucoside and quercetin-3-rhamnoside were lower compared to its aglycone quercetin.

Glycosylation seemed to play an important role in the uptake of oleic acid. The same trend was also observed for isorhamnetin-3-glucoside and kaempferol-3-rutinoside, both showed a lower uptake of oleic acid than its aglycone isorhamnetin and kaempferol. Quercetin-3-glucoside showed a higher enhancement of oleic acid uptake compared to quercetin-3-rhamnoside at 10 µM (13.1% ± 3.5% and 5.0% ± 6.0%, respectively) in the skeletal muscle cells (Figure 3B). The sugar moiety attached to flavonoid aglycones seems therefore to influence the oleic acid uptake in both the skeletal muscle cells and HepG2-cells. Naringenin, isorhamnetin and kaempferol showed a high response in oleic acid uptake in the skeletal muscle cells, with kaempferol as the most potent one, with an increase of 25.0% ± 3.0% at 10 µM. Naringenin and kaempferol have identical A and B rings, but differs in the C-ring (Figure 1). The chemical characteristics such as the number and position of hydroxyl groups, the molecular weight, interlinkage between B and C rings as well as the saturation of double bonds in the rings may have a slight effect on the efficacy of the compound. Epicatechin and catechin appeared almost equally active at the same molar concentration in the skeletal muscle cells (Figure 3C). In the HepG2-cells epicatechin showed a significant higher increase of oleic acid compared to catechin at 1 and 10 µM. The same phenomenon was also observed for the isomers neochlorogenic acid and chlorogenic acid that appeared almost equally active at the same molar concentration in the skeletal muscle cells. At the highest concentration tested chlorogenic acid showed an enhancement of oleic acid uptake of 17.3% ± 2.5% in the skeletal muscle cells and 25.6% ± 4.6% in the HepG2-cells. Chlorogenic acid is present in high content in elderflowers [21], and might be an important contributor to the stimulation of oleic acid uptake in both the skeletal muscle cells and HepG2-cells of elderflowers. In the HepG2-cells, epicatechin showed a significant higher increase of oleic acid uptake compared to catechin at 1 and 10 µM. 3-Hydroxyphenylacetic acid, 3,4-dihydroxyphenylacetic acid and 4-methylcatechol showed a small but significant increase in oleic acid uptake at 10 µM (Figure 3C).

### 2.3. α-Amylase and α-Glucosidase Inhibitory Activity

α-Amylase and α-glucosidase are key enzymes involved in starch breakdown and intestinal glucose absorption. α-Glucosidase acts during the final step in the digestive process of carbohydrates, as it catalyzes the cleavage of glucose from oligosaccharides and disaccharides. The inhibition of these enzymes can slow down the overall absorption rate of glucose into the blood and has proved to be a good strategy to reduce postprandial plasma glucose levels and suppress postprandial hyperglycemia, which might help to prevent the onset of diabetes or long-term diabetic complications [31]. In this study, crude extracts, constituents and metabolites from elderflowers were investigated for their inhibition of α-amylase and α-glucosidase activity. Elderflower extracts have not been tested for inhibition of α-amylase or α-glucosidase previously. All crude extracts had the capacity to inhibit the yeast α-glucosidase enzyme and the porcine pancreatic α-amylase (Table 1).

Among the crude extracts, the 96% EtOH extract showed the strongest inhibition of both α-amylase (IC_50_ 2.8 ± 1.1 µg/mL) and α-glucosidase (IC_50_ 4.8 ± 0.5 µg/mL) as compared with acarbose used as control compound (IC_50_ 84.7 ± 3.8 µg/mL for α-glucosidase, IC_50_ 73.3 ± 4.3 for α-amylase). Pectic polysaccharides have been shown in many studies to inhibit α-glucosidase and α-amylase, decreasing blood glucose levels and act as potent hypoglycemic agents [32,33,34]. However, the mechanism of action is unclear. The water extracts from elderflowers contain large amounts of polysaccharides as shown in previous studies [5,6]. The 50 °C and 100 °C water extracts showed potent α-glucosidase and α-amylase inhibitory activities, and both were more active than acarbose. The α-glucosidase and α-amylase inhibitory activities of elderflower constituents and the metabolites are shown in Table 2.

Quercetin (IC_50_ 2.6 ± 0.9 µM) and kaempferol (IC_50_ 4.5 ± 1.2 µM) were the most active α-glucosidase inhibitors followed by naringenin (IC_50_ 7.5 ± 1.1 µM). Regarding to α-amylase inhibitory activity quercetin, kaempferol and naringenin possessed relatively high inhibitory effects, as well, with IC_50_ values of 2.1 ± 0.5, 3.6 ± 1.1 and 6.2 ± 0.7 µM, respectively. In our experiments, the tested compounds and acarbose are considerably more active than previously reported [35,36]. The discrepancy may be due to differences in the experimental setup or to different enzyme sources. The inhibitory potential of quercetin against α-glucosidase and α-amylase was stronger than quercetin-3-glucoside, quercetin-3-rhamnoside and rutin. Thus illustrating that the glycosylation of flavonoids decrease the inhibitory effect against α-amylase and α-glucosidase. The same findings are also confirmed in other studies [35,37]. The same phenomenon was also observed for isorhamentin and its glycosides and kaempferol and its glycosides. It was suggested by Xiao et al. [35] that glycoside substitution might lead to steric hindrance which weakens the binding interaction between flavonoids and the α-amylase and α-glucosidase. However, a study performed by Manaharan, et al. [38] showed that myricetin 3-*O*-rhamnoside showed stronger inhibition of α-amylase than of myricetin. Overall, it is difficult to draw general or universally applicable comments regarding the impact of glycosylation on flavonoids’ biological benefits. Chlorogenic acid and neochlorogenic acids showed high α-amylase and α-glucosidase inhibitory activities in fair accordance with previously study [37]. Catechin was less active as α-amylase and α-glucosidase inhibitor compared to the isomer epicatechin, indicating that minor structural differences might influence their ability to inhibit α-amylase and α-glucosidase. A previous study has also shown that the inhibitory activity of epicatechin was higher than catechin [39]. However Ishikawa, et al. [40] found the inhibitory activity of catechin to be higher than epicatechin against α-glucosidase. The degradation products 3-hydroxyphenylacetic acid, 3,4-dihydroxyphenylacetic acid and 4-methylcatechol exhibited potent α-amylase and α-glucosidase inhibition. This α-amylase and α-glucosidase inhibitory action seen in elderflowers may contribute to decreasing the level of blood glucose, resulting in a significant reduction in the incidence of chronic vascular complication in diabetic patients.

### 2.4. Free Radicals and Antioxidant Activities

Oxidative stress is a physiological state which has been suggested to be an important factor for the development of many diseases including diabetes, cardiovascular diseases, stroke and neurodegenerative disorders [15,16]. Scavenging of DPPH radical was used to measure the antioxidant activity of the elderflower extracts, constituents and metabolites (Table 1 and Table 2). The 96% EtOH extract showed the highest DPPH radical scavenging activities with IC_50_ value of 9.2 ± 0.9 µg/mL. This might suggest that 96% EtOH extract has a higher selectivity for phenolics and antioxidant components. The DCM extract of elderflower was inactive as radical scavenger. Previous studies have shown that elderflower extracts possessed strong antioxidant activity measured by ferric reducing antioxidant power (FRAP), 2,2′-azino-bis(3-ethylbenzothiazoline-6-sulfonic acid (ABTS) and DPPH assays [21,41]. The IC_50_ values of kaempferol and kaempferol-3-rutinoside as DPPH-radical scavengers were 10.6 ± 3.9 µM and 30.6 ± 3.9 µM, respectively. The glycosylation of flavonoids obviously reduced the DPPH radical scavenging activity. The same phenomenon was observed with quercetin and its glycosides with their IC_50_ values in this order quercetin (9.3 ± 1.5 µM) > quercetin-3-glucoside (17.6 ± 3.2 µM) > quercetin-3-rhamnoside (19.1 ± 2.1 µM) > rutin (22.5 ± 1.6 µM). Our results are consistent with previous reports on antioxidant activity of flavonoids being dependent on the number and position of substituted OH groups and with the presence of sugar residues [35]. Monophenolic compounds were generally weaker as radical scavengers compared to the polyphenols. 3-Hydroxyphenylacetic acid (IC_50_ 125.3 ± 4.8 µM), 3,4-dihydroxy phenyl acetic acid (IC_50_ 115.9 ± 1.4 µM) and 4-methylcatechol (IC_50_ 40.5 ± 3.6 µM) showed moderate DPPH radical scavenging activity, with 4-methylcatechol as the most active one. The DPPH activity of the degradation products has not been reported in previous literature.

The inhibitory potency of the extracts and constituents from elderflowers towards peroxidation of linoleic acid catalyzed by soybean 15-LO was studied (Table 1 and Table 2). 15-LO has been shown to be involved in a number of diseases such as cancer, psoriasis and diabetes (both type I and II) [42]. Inhibition of 15-LO is of interest, as the enzyme has been proposed to have a role in the oxidation of low density lipoprotein (LDL), a process which is believed to be an important step in the development of atherosclerosis, diabetes and cardiovascular diseases [42]. The DCM extract was inactive as inhibitors of 15-LO, but the 96% (IC_50_ 17.9 ± 3.6 µg/mL) and 50% EtOH (IC_50_ 24.4 ± 3.1 µg/mL) extracts showed higher activity compared to the positive control quercetin (IC_50_ 29.3 ± 1.9 µg/mL). The 50 °C (IC_50_ 126.6 ± 3.9 µg/mL) and 100 °C water (IC_50_ 75.9 ± 6.5 µg/mL) extracts were moderately active as 15-LO inhibitors. The enzyme inhibition of elderflower crude extracts is not previously reported. The elderflower constituents possessed high 15-LO inhibitory ability, with kaempferol (93.7 ± 3.7 µM) and rutin (99.3 ± 1.1 µM) being the most active ones. Rutin has previously been reported to be a good 15-LO inhibitor, in good accordance with our findings [43]. Quercetin which is a constituent in elderflower, used as a positive control in this assay, showed high 15-LO inhibition (95.9 ± 1.3 µM). The aglycone isorhamnetin, kaempferol and quercetin showed strong activity, while lower activities were observed for the glycosylated flavonoids. The reduced 15-LO inhibitory activity of glycosylated flavonoids has been previously reported [44]. Naringenin, epicatechin, catechin were less potent than quercetin. The lack of 2,3-double bond in the C-ring of the flavonoid seemed to be important for the activity, as well. 3-Hydroxyphenylacetic acid, 3,4-dihydroxyphenylacetic acid, 4-methylcatechol and benzoic acid were less active. It seems that the potent 15-LO inhibition observed for the crude extracts could not be ascribed to any of the tested compounds. There might be unknown potent 15-LO inhibitors in the extracts or the effect are caused by synergistic effects.

XO is thought to be one of the main mechanisms of ROS production in diabetes [17,45]. The inhibitory effect of elderflower extracts, constituents and metabolites towards the superoxide-producing enzyme XO from cow’s milk are shown in Table 1 and Table 2. The EtOH extracts possessed modest activity in the XO inhibition assay, with the 96% EtOH extract as the most active one (IC_50_ 59.3 ± 6.3 µg/mL). The DCM extract was inactive towards XO. Among the flavonoids kaempferol (1.8 ± 0.3 µM), quercetin (IC_50_ 2.3 ± 0.3 µM) and isorhamnetin (IC_50_ 2.8 ± 1.1 µM) possessed the strongest XO-inhibition. The XO-inhibition of kaempferol and isorhamnetin is in accordance with previous investigations [46,47]. The presence of a hydroxyl groups at C-3’, C-3, C-5 and C-7 is associated with high XO inhibition [48]. Chlorogenic acid (24.2 ± 5.3 µM) and neochlorogenic acid (26.2 ± 3.1 µM) were both potent inhibitors of XO, which are in fair accordance with previous results [49,50]. Epicatechin and catechin did not inhibit XO up to the highest concentration tested (167 µM). The degradation products 3-hydroxyphenylacetic acid, 3,4-dihydroxyphenylacetic acid, 4-methylcatechol and benzoic acid were all inactive at the highest concentration tested (167 µM).

## 3. Materials and Methods

### 3.1. Plant Material

Certified ecologically cultivated flowers of *Sambucus nigra* L., harvested in Norway October 2012, were purchased from Odins Marked, Oslo, Norway (org. No.: 876905892) in November 2012. The plant material was freeze-dried, pulverized in a mechanical grinder (0.4 mm) and stored dry in closed vessels below 5 °C. A voucher specimen (nr: EF1020) is deposited in the Pharmacognosy section, School of Pharmacy, University of Oslo, Norway.

### 3.2. Chemicals

Chlorogenic acid, neochlorogenic acid, epicatechin, catechin, kaempferol, kaempferol-3-rutinoside, 3-hydroxyphenylacetic acid, 3,4-dihydroxyphenylacetic acid, isorhamnetin-3-rutinoside, naringenin, isorhamnetin, rutin, quercetin, quercetin-3-rhamnoside, quercetin-3-glucoside, 4-methylcatechol, benzoic acid, DMSO-*d*_6_, CD_3_OD, linoleic acid, 15-lipoxygenase (15-LO) from soybeans, 4-nitrophenyl α-d-glucopyranoside (PNP-G), 2-chloro-4-nitrophenyl-α-d-maltotrioside (CNPG3), xanthine oxidase from bovine milk, hypoxanthine, 1,1-diphenyl-2-picrylhydrazyl (DPPH) radical, acarbose, 22-*S*-hydroxycholesterol (22-SHC) and bovine serum albumin (BSA) (essentially fatty acid-free) were purchased from Sigma-Aldrich (St. Louis, MO, USA). T0901317 was obtained from Cayman Chemicals (Ann Arbor, MI, USA). Dulbecco’s modified Eagle’s medium (DMEM-Glutamax, 5.5 mM), DMEM, fetal bovine serum, Ultroser G, penicillin–streptomycin–amphotericin B, and trypsin-EDTA were obtained from Gibco, Life Technologies (Paisley, UK). [^14^C(U)]-glucose (1 μCi/mL, 100 μM) and [^14^C]-oleic acid (37 kBq, 100 µM) were purchased from ARC (American Radiolabeled Chemicals, St. Louis, MO, USA). Corning CellBIND tissue culture plates were obtained from Corning Life-Sciences (Schiphol-Rijk, The Netherlands). The protein assay reagent was obtained from BioRad (Copenhagen, Denmark). All other reagents were of the highest purity available.

### 3.3. Extraction

Elderflowers were freeze-dried, pulverized and extracted with dichloromethane followed by 96% EtOH on an accelerated solvent extractor (Dionex, Sunnyvale, CA, USA). The residue was further extracted with 50% EtOH at 70 °C, and with water at 50 °C and 100 °C. The extractions were performed at 1500 psi, with 5 min heating, 5 min static time, and a 60 s purge for a total of three cycles.

### 3.4. NMR

^1^H nuclear magnetic resonance (NMR) experiments were conducted on a DPX 300 or AVII 400 instrument (Bruker, Rheinstetten, Germany) with CD_3_OD or DMSO-*d*_6_ as solvents and tetramethylsilane (TMS) as reference.

### 3.5. Culturing of Human Myotubes

Satellite cells were isolated from the *Musculus obliquus internus abdominis* of four healthy donors, age 34.8 (±19) years, body mass index 22.9 (±2.7) kg/m^2^, fasting glucose 4.9 (±0.6) mM, insulin, plasma lipids and blood pressure within normal range and no family history of diabetes. The muscle biopsies were obtained with informed consent and approval by the National Committee for Research Ethics (Oslo, Norway). The cells were cultured on 96-well CellBIND microplates DMEM-Glutamax (5.5 mM glucose), 2% fetal bovine serum, 2% Ultroser G, penicillin (100 units/mL), streptomycin (100 μg/mL) and amphotericin B (1.25 μg/mL) for proliferation. At 70%–80% confluence, the growth medium was replaced by DMEM-Glutamax (5.5 mM glucose) supplemented with 2% fetal bovine serum, penicillin (100 units/mL), streptomycin (100 μg/mL), amphotericin B (1.25 μg/mL) and insulin (25 pM), to induce differentiation. The cells were cultured in humidified 5% CO_2_ atmosphere at 37 °C, and the medium was changed every 2–3 days. Experiments were performed after 7 days of differentiation. 22-*S*-hydroxycholesterol (22-SHC) (10 µM) was used as positive control in the glucose uptake assay [50]. The positive control used in the oleic acid assay is the synthetic liver X receptor (LXR) agonist T0901317 (10 µm) [51].

### 3.6. Culturing of HepG2-Cells

The human hepatoblastoma cell line HepG2 (HB-8065, ATCC, Manassas, VA, USA) was cultured in DMEM-Glutamax (5.5 mM glucose) supplemented with 10% fetal bovine serum, streptomycin (100 μg/mL) and penicillin (100 units/mL) at 37 °C in 5% CO_2_.

### 3.7. Glucose and Oleic Acid Uptake

Cells were exposed to test samples solved in 0.1% DMSO that were added directly to the medium for 2 days for myotubes and 24 h for HepG2 cells. Thereafter, cells were exposed to [^14^C(U)]-glucose (1 μCi/mL, 100 μM) or [^14^C]-oleic acid (37 kBq, 100 µM) for 4 h. A 96-well UniFilter-96 GF7B microplate (Perkin Elmer, Waltham, MA, USA) was mounted on top of the CellBIND plate (Corning Life-Sciences (Schiphol-Rijk, The Netherlands) and CO_2_ production was measured. After incubation the cells were washed twice with ice-cold phosphate buffered saline (PBS), lysed in 0.1 M NaOH, and radioactivity measured by liquid scintillation counting [52]. The protein content of each sample was determined and glucose uptake was calculated using protein levels for standardization [53].

### 3.8. α-Glucosidase Inhibitory Activity

The α-glucosidase inhibitory activity of the extracts was assessed according to the method described by Ranilla, et al. [54]. Baker’s yeast α-glucosidase (EC 3.2.1.20) was purchased from Sigma-Aldrich Chemie GmbH (Steinheim, Germany). Acarbose was used as positive control.

### 3.9. α-Amylase Inhibitory Activity

The α-amylase assay was performed as described by Gella, et al. [55]. Porcine pancreatic α-amylase (EC 3.2.1.1) was purchased from Sigma-Aldrich Chemie GmbH. Acarbose was used as positive control.

### 3.10. DPPH Radical Scavenging

Scavenging activity towards the DPPH radical was carried out as previously described [56]. Test substances were dissolved in DMSO or water. Values were corrected for absorbance of the test substances. Quercetin was used as a positive control.

### 3.11. Inhibition of 15-Lipoxygenase

Soybean lipoxygenase with linoleic acid as the substrate was used to measure inhibition of 15-LO. Test substances were dissolved in DMSO or water, and the assay was carried out as previously described [36]. Quercetin was used as a positive control.

### 3.12. Inhibition of Xanthine Oxidase

The XO inhibitory activity with hypoxanthine as the substrate was carried out previously described [36]. Test substances were dissolved in DMSO or water. Quercetin was used as a positive control.

### 3.13. Statistical Analysis

Data and figures for glucose and oleic acid uptake are given as mean (±SEM) from *n* = number of separate experiments. At least three replicates were included in each experiment. Comparisons of different treatments were evaluated by two-tailed and paired Student’s *t*-test. Samples for DPPH, 15-LO, α-glucosidase and α-amylase assays were analyzed in triplicate and results are given as averages ± SD. Student *t*-test was used for testing a hypothesis on the basis of a difference between two means (test samples vs. DMSO control) and determines whether any of those means are statistically significant different from each other. *p* < 0.05 vs. DMSO control was considered significant in all the assays. Statistical analyses were performed using GraphPad Prism 5.0 for Windows (GraphPad Software, San Diego, CA, USA).

## 4. Conclusions

The main findings from these studies are that elderflower extracts, their constituents and the corresponding flavonoid metabolites showed a major effect on the enhancement of glucose uptake and oleic acid uptake in human liver cells and human skeletal muscle cells. Elderflowers might exert their antidiabetic effects through several active compounds, which act on different cellular targets in the liver and the muscle. This is the first account demonstrating the ability of elderflower to enhance glucose and oleic acid uptake in human skeletal muscle cells and in human liver cells. Kaempferol and its glycosides and quercetin and its glycosides showed strong stimulation of glucose and oleic acid uptake. However, due to the complex bioavailability of flavonoids, it is difficult to know the most relevant substances after intake of elderflowers in humans. Elderflower constituents and metabolites also act as strong antioxidants and might play an important role in the controlling of postprandial hyperglycemia by strong inhibition of α-glucosidase and α-amylase. The antidiabetic properties found in phenolics from elderflower increase the nutritional value of this plant as a functional food against diabetes.

## Figures and Tables

**Table 1:** Scavenging of 1,1-diphenyl-2-picrylhydrazyl (DPPH) radical, 15-lipoxygenase (15-LO), xanthine oxidase (XO), α-glucosidase and α-amylase inhibitory activity of elderflower crude extracts. IC_50_ values ± standard deviation (SD) are shown.

Elderflower Extract	DPPH (µg/mL)	15-LO (µg/mL)	XO (µg/mL)	α-Glucosidase (µg/mL)	α-Amylase (µg/mL)
DCM	>167	125.9 ± 3.9	>167	105 ± 5.6	103 ± 5.9
96% EtOH	9.2 ± 0.9	17.9 ± 3.6	59.3 ± 6.3	4.8 ± 0.5	2.8 ± 1.1
50% EtOH	20.2 ± 3.9	24.4 ± 3.1	79.5 ± 4.1	8.9 ± 1.1	3.1 ± 1.3
50 °C Water	68.9 ± 2.3	126.6 ± 3.9	156.5 ± 5.3	78.9 ± 5.8	71.8 ± 4.1
100 °C Water	32.0 ± 2.9	75.9 ± 6.5	135.6 ± 7.8	65.3 ± 4.6	66.2 ± 5.6
Quercetin (control)	2.8 ± 0.3	29.3 ± 1.9	0.7 ± 0.2	nt	nt
Acarbose (control)	nt	nt	nt	84.7 ± 3.8	73.3 ± 4.3

nt: Not tested. DCM: dichloromethane.

**Table 2:** Scavenging of DPPH radical, 15-LO, XO, α-glucosidase and α-amylase inhibitory activity of phenolic compounds from elderflower. IC_50_ values ± SD are shown.

Test Compound	DPPH ^1^ (µM)	15-LO ^1^ (µM)	XO ^1^ (µM)	α-Glucosidase ^2^ (µM)	α-Amylase ^3^ (µM)
**Phenolic Compounds**					
Quercetin ^1^	9.3 ± 1.5	95.9 ± 1.3	2.3 ± 0.3	2.6 ± 0.9	2.1 ± 0.5
Quercetin-3-glucoside	17.6 ± 3.2	102.3 ± 5.3	105.9 ± 5.3	4.1 ± 1.9	3.0 ± 1.2
Quercetin-3-rhamnoside	19.1 ± 2.1	108.4 ± 4.6	104.6 ± 4.6	3.9 ± 1.4	3.5 ± 0.9
Rutin (Quercetin-3-rutinoside)	22.5 ± 1.6	99.3 ± 1.1	42.9 ± 2.9	4.6 ± 2.3	4.1 ± 0.8
Kaempferol	10.6 ± 3.9	93.7 ± 3.7	1.8 ± 0.3	4.5 ± 1.2	3.6 ± 1.1
Kaempferol-3-rutinoside	30.6 ± 3.9	108.7 ± 5.6	63.8 ± 2.1	23.9 ± 1.1	19.1 ± 0.5
Isorhamnetin	63.3 ± 2.3	103.1 ± 2.4	2.8 ± 0.7	8.1 ± 3.1	7.5 ± 0.9
Isorhamnetin-3-rutinoside	85.0 ± 2.1	115.3 ± 6.2	125.0 ± 3.9	25.2 ± 2.9	26.2 ± 0.7
Naringenin	23.3 ± 1.4	124.1 ± 3.5	95.1 ± 4.5	7.5 ± 1.1	6.2 ± 1.1
Catechin	19.0 ± 1.1	128.1 ± 5.9	>167	18.5 ± 2.2	14.1 ± 0.8
Epicatechin	15.6 ± 2.3	115.6 ± 7.9	>167	12.1 ± 2.3	9.7 ± 2.1
Caffeic acid	90.3 ± 4.3	125.9 ± 4.7	107.3 ± 3.2	18.5 ± 0.9	13.9 ± 0.7
Chlorogenic acid	17.5 ± 3.9	106.2 ± 2.3	24.2 ± 5.3	10.5 ± 2.1	9.1 ± 1.1
Neochlorogenic acid	19.6 ± 1.6	115.1 ± 5.8	26.2 ± 3.1	13.1 ± 1.3	15.4 ± 3.2
**Metabolites**					
Benzoic acid	145.3 ± 5.8	137.6 ± 6.5	>167	128.9 ± 3.8	124.1 ± 5.3
3-Hydroxyphenylacetic acid	125.3 ± 4.8	133.9 ± 5.8	>167	68.9 ± 3.8	44.8 ± 5.3
3,4-Dihydroxyphenylacetic acid	115.9 ± 1.4	135.5 ± 7.3	>167	78.5 ± 1.6	74.9 ± 1.7
4-Methylcatechol	40.5 ± 3.6	129.0 ± 5.2	>167	98.9 ± 3.5	94.8 ± 6.8

^1^ Quercetin was used as positive control in DPPH, 15-LO and XO assays; ^2^ Acarbose was used as a positive control (IC_50_ 131.2 ± 9.3 µM); ^3^ Acarbose was used as a positive control (IC_50_ 113.5 ± 4.6 µM).
